# Methylphenidate use and school performance among primary school children: a descriptive study

**DOI:** 10.1186/s12888-017-1279-1

**Published:** 2017-03-29

**Authors:** Jurjen van der Schans, Rukiye Çiçek, Sefike Vardar, Jens HJ Bos, Tjalling W de Vries, Pieter J Hoekstra, Eelko Hak

**Affiliations:** 1 Groningen Research Institute of Pharmacy, PharmacoTherapy, -Epidemiology & -Economics, University of Groningen, Antionius Deuginslaan 1, 9713 AV Groningen, the Netherlands; 2 Department of Pediatrics, Medical Center Leeuwarden, Henri Dunantweg 2, 8934 AD Leeuwarden, the Netherlands; 3 Department of Psychiatry, University of Groningen, University Medical Center Groningen, Hanzeplein 1, 9713 GZ Groningen, the Netherlands; 4 Department of Epidemiology, University Medical Center Groningen, Hanzeplein 1, 9713 GZ Groningen, the Netherlands

**Keywords:** Methylphenidate, Attention-deficit/hyperactivity disorder, School performance, Pediatrics

## Abstract

**Background:**

There is no conclusive evidence that stimulants have beneficial effects on major associated outcome parameters, particularly school performance. We assessed the differences in school performance among children using methylphenidate at the end of primary school in relation to various parameters of methylphenidate use.

**Methods:**

We linked children from a pharmacy prescription database with standardized achievement test results at the end of primary school. We explored differences in test scores between current methylphenidate users versus never users and methylphenidate users who stopped treatment at least 6 months before the test, early versus late starters, different dosage of methylphenidate, and concurrent antipsychotic or asthma treatment.

**Results:**

Out of the 7736 children, 377 (4.9%) children were treated with methylphenidate at the time of the test. After adjusting for confounders the methylphenidate users (532.58 ± .48) performed significantly lower on the test than never users (534.72 ± .11). Compared with late starters of methylphenidate treatment (536.94 ± 1.51) we found significantly lower test scores for the early starters (532.33 ± .50).

**Conclusion:**

Our study indicates that children using methylphenidate still perform less at school compared to their peers. Our study also suggests that earlier start of methylphenidate treatment is associated with a lower school performance compared to children starting later with the treatment. This result could either indicate a limited effect of long term treatment or a more strongly affected group of early starters.

## Background

Attention-deficit/Hyperactivity disorder (ADHD) is the most commonly diagnosed psychiatric disorder of childhood and is prevalent among approximately 3–5% of school-aged children [1]. The negative impact of ADHD on school performance is profound. Children with ADHD have been shown to have lower grades, lower mathematics and reading achievement scores, and a higher rate of school dropout and grade retention [2–5]. Treatment of ADHD usually consists of a multi-model approach in which combinations of pharmacological and non-pharmacological options are being used. Besides the non-pharmacological options like behavioral therapy (for both child and parents), cognitive training, neurofeedback, psychoeducation, and dietary interventions [6], stimulants are the first drug of choice in the management of ADHD. In the Netherlands methylphenidate is most frequently used [7]. In a recent systematic review on the efficacy of methylphenidate the authors concluded that methylphenidate might have a positive effect on teacher-reported ADHD symptoms and teacher-reported general behavior, but quality of the reviewed studies was low and efficacy of psychostimulants was questioned [8]. However, as stated in a commentary on this study, the study’s methods has been criticized its outcome heavily contradicts the wealth of available studies [9]. In general, most studies have concluded that the use of methylphenidate in children and adolescents with ADHD also has a positive effect on school performance when looking at different medication types of methylphenidate versus controls [10–13], and differences in adherence [14]. These results were confirmed by the meta-analysis of Prasad et al. in which the authors concluded that medication used in ADHD treatment could improve academic achievement [15]. All in all, however, the long term effectiveness of stimulants on school performance remains unclear in spite of evidence of their short term efficacy in relieving the core symptoms of ADHD and the positive effect on school performance in controlled trial settings. Moreover, it is questioned whether the efficacy of methylphenidate on school performance holds in real-life treatment settings, outside of formal trials, especially on the long term. Therefore, population based studies, especially focusing on long term effectiveness are warranted.

In this study we explored the differences in school performance at the end of primary school between children using methylphenidate and their peers. In addition, we assessed differences in school performance among various relevant subgroups and characteristics of medication use.

## Methods

### Study setting

For this cohort study an analysis of the University of Groningen IADB.nl pharmacy prescription database was performed. The IADB database is a longitudinal pharmacy-dispensing database with detailed patient-based drug prescription data from 1994 till 2012 from approximately 600.000 patients in the Netherlands. Prescription rates have been found to be representative of the Netherlands as a whole, and the database has been widely used for research [16]. Patient anonymity is guaranteed by the use of an unique anonymous identifier, hence the ethical approval for observational studies with data from the IADB has been waived. Dutch patients usually register at a single community pharmacy and therefore this pharmacy can provide an almost complete listing of the subject’s prescribed drugs [16].

### Study population

**Fig. 1:**
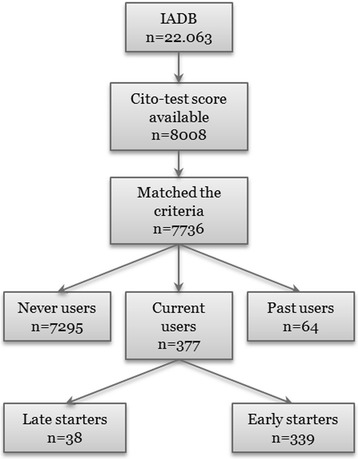
Flow chart of study population. Abbreviations: IADB, InterActive DataBase; ADHD, Attention-Deficit/Hyperactivity Disorder; MPH, Methylphenidate

Our study population consisted of 22,063 children born between 1996 and 2001 (Fig. 1). By using a personal identification number unique to every citizen, a linkage with data from Statistics Netherlands (CBS) was performed. Statistics Netherlands was responsible for performing the linkage between the two datasets and removed all identification information from the dataset, hence researchers were unable to identify patients. The final study population comprised all children whose full data were available (*n* = 7736).

### Outcome measure

In the Netherlands, all children at the end of primary education around the age of 12 choose which type of secondary education is the most appropriate for them. At the end of primary school, the Central Institute for Test Development (in Dutch: Cito-) test is performed yearly in February in about 85% of the Dutch primary schools [https://www.centraleeindtoetspo.nl/media/uploads/files/cvte_tabellen_schaalscore_per_schooltype_en_brugklastype_2015.pdf]. The Cito-test score is a figure between 501 and 550, based on a transformation of the number of correct answers for two hundred multiple choice questions covering language, arithmetic/mathematic, and general study skills. Because of this transformation we were able to compare the scores from year to year despite difference in difficulty of the tests. The score is an indicator for the learning achievement of a child; indirectly it also is an indicator for intelligence, motivation, concentration, and drives to learn. For the reasons mentioned above the Cito-test is used as an independent, standardized, educational achievement test which indicates the most suitable type of secondary education(≤523: secondary education leading to vocational education level 3; 524–528: level 2; 529–536: level 1; 537–544: secondary education leading to higher professional education; ≥545 secondary education leading to university).

### Methylphenidate exposure

Methylphenidate exposure was defined as having at least one dispensed prescription for methylphenidate in the period 2001 to 2012. We included children with a follow-up period from the date of the first dispensing of a prescription to at least the year the Cito-test was taken. Children were only included in the study when they were present in the database for at least 1 year before initiation of methylphenidate treatment. We excluded children with the date of the first dispensing of a prescription after March in the year the Cito-test was taken. In addition, children who took the Cito-test twice were also excluded. Non-exposure (never users) was defined as having no prescription of methylphenidate, and no prescription of dexamphetamine or atomoxetine due to their association with ADHD. The diagnostic status of the children was not available.

### Methylphenidate users

We considered children to have been treated on their test day (February) if they had at least one dispensed prescription for methylphenidate in the year the Cito-test was taken from January 1^st^ to March 31^st^. We defined this treatment group as current users.

We compared current with past users, children who have had at least one dispensed prescription of methylphenidate but no prescription of methylphenidate in the 6 months before the Cito-test.

To estimate the influence of confounding by indication we further assessed the association between school performance and different patterns of methylphenidate use by comparing early versus late start of treatment, current versus past users, different dose regimens and co-occurring conditions such as psychiatric disorders treated with antipsychotics and atopic diseases which may all influence school performances [17]. Children with ADHD are more commonly affected with atopic diseases like eczema, asthma, and allergic rhinitis [18, 19].

### Start of treatment

The start of treatment for each child was defined by the date of the first dispensing of a prescription for methylphenidate within the study period. We compared school performance within the current users group according to methylphenidate treatment start, e.g. early vs. late start of treatment. We used February in the year of the Cito-test as reference date and we defined starting the treatment within 12 months (late start) and later than 12 months (early start) before this reference date.

### Dosage of methylphenidate treatment

We compared school performance within the current users group according to their different dosage of methylphenidate treatment. The dosage was defined as the number of Defined Daily Dose (NDDD) per day, which we calculated by dividing the total number of DDD’s prescribed for the drug by the theoretical number of days the drug would be used. To compare different methylphenidate dosage treatment groups we classified the current users with prescriptions for methylphenidate in different subgroups according to NDDD’s per day. Since all children take the Cito tests around the same age we assumed the dosage was not dependent on body weight, but related to severity.

### Concurrent treatment

We also considered concurrent drug use of other psychotropic drugs. Concurrent drug use was defined as at least 1 prescription for any psychotropic drug in the year the Cito-test was taken from January 1st to March 31st in addition to at least 1 prescription for methylphenidate in the same period. Other psychotropic drugs were defined as all psychotropic drugs other than methylphenidate.

In addition, we also sought to determine concurrent use of medication for atopic diseases, i.e., inhaled corticosteroids, short acting beta agonists, nasal steroids, and dermatological corticosteroids. Concurrent use of medication for atopic diseases was defined as having at least 2 prescriptions within a 12-month period in the year the Cito-test was taken, in addition to having at least 1 prescription from January 1^st^ to March 31^st^.

### Potential covariates

Demographic data on every child during the study period, including sex, ethnicity, presence of single parenthood, and household income was obtained from the CBS. We used data registered to 2012, which represented the most recent years of the IADB prescription data available at the time of our study. We used the household income data of the year 2011, which represents the halfway of the period in which the Cito-test scores were available. Also, we used the type of household information of the year the Cito-test was taken. As all children were 12 or 13 years old we did not include birth year as a potential confounding factor.

We classified ethnicity as Dutch or non-Dutch, based on the mother’s country of birth. Household income was defined as the standardized disposable household income in percentiles. We assigned household income to subgroups according to household income as follows: among the high-income group we considered the highest 20% household incomes. These households have an income of at least 30,000 euros. The low-income group included the lowest 40% household incomes with an income around 18,550 euros. The intermediate-income group considered the remaining 40% household incomes.

### Statistical analysis

The frequencies of the baseline characteristics were compared by using Pearson chi-squared test. We also described the characteristics of methylphenidate treatment, e.g. start of treatment, dosage of treatment, and concurrent use of antipsychotics or asthma medication. We described crude mean Cito-test scores and tested for significance difference with analysis of variance (ANOVA). Then we tested the interaction terms between covariates and methylphenidate exposure by using backward elimination to exclude non-contributable confounding (*p* value > .05) from the prediction model to measure whether the association between methylphenidate use and Cito-test scores differed. An analysis of covariance (ANCOVA) was subsequently conducted to test whether there was a statistically significant difference in school performance measured by Cito-test scores between the methylphenidate treatment groups after controlling simultaneously for the effects of confounding. Finally, we performed ANCOVA analyses to test for significant differences in school performance between the different treatment groups. In this study only children with full data on all of the included variables were analyzed. Cohen’s d was calculated to determine the standardized difference between two means for the significant findings (Cohen’s d ≥ 0.20; small difference; ≥0.50: moderate difference; ≥0.80 large difference). All analyses were conducted using Statistical Package for Social Sciences (SPSS) version 22 and a two sided *p* value <0.05 was considered to be statistically significant.

## Results

Characteristics between never users of methylphenidate and current users during the time of Cito-test are shown in Table 1. Baseline characteristics among our study population varied for sex, ethnicity and household income. Single parenthood was equally distributed over the current and never users.

**Table 1:** Comparison of the characteristics between never users and current users

	Never users, n (%)	Current users, n (%)	*p* value (Chi^2^)
Total	7295 (100)	377 (100)	
Sex			<.05*
Boys	3275 (44.9)	298 (79.0)	
Girls	4020 (55.1)	79 (21.0)	
Ethnicity			<.05*
Dutch	6407 (87.8)	358 (95.0)	
non-Dutch	888 (12.2)	19 (5.0)	
Parent Household			.656
Two-parent household	5987 (82.1)	306 (81.2)	
single-parent household	1308 (17.9)	71 (18.8)	
Household income			<.05*
low	3010 (41.3)	171 (45.4)	
intermediate	3176 (43.5)	166 (44.0)	
high	1109 (15.2)	40 (10.6)	
Treated concurrently with			
Asthma medication	100 (1.4)	11 (2.5)	<.05*
Antipsychotics		19 (4.3)	
Only MPH users		347 (78.7)	
Start of MPH treatment			
Late ≤12 months		38 (10.1)	
Early >12 months		339 (89.9)	
Dosage of MPH treatment			
≤ 0.999 NDDD/Day		202 (53.6)	
> 0.999 NDDD/Day		132 (35.0)	

Abbreviations: n, sample size; NDDD, number of Defined Daily Dose

* Significant at .05 level

We only found sufficient numbers of medication users with regard to antipsychotics in the other psychotropic medication group, and asthma medication in the atopic medication group. Therefore we could only compare concurrent use of antipsychotics with methylphenidate use and concurrent use of asthma medication with methylphenidate use.

Table 2 shows the baseline characteristics of the study population and results of univariate analyses of the possible confounders in relation to Cito-test scores.

The difference in Cito-test scores between the household income categories tended to significance after excluding the non-significant interaction terms (*p* value .101 from.153).

**Table 2:** Baseline characteristics of the study population and results of univariate analyses of the possible confounders in relation to Cito-test scores

	Never users M ± SD	Current users M ± SD	Subgroups *p* value	interaction *p* value
Total	534.71 ± 9.47	532.80 ± 9.44	<0.001***	
Sex				.020*
Boys	535.25 ± 9.42	533.60 ± 9.26	0.004**	
Girls	534.27 ± 9.49	529.75 ± 9.55	<0.001***	
Ethnicity				.296
Dutch	535.09 ± 9.33	532.82 ± 9.41	<0.001***	
Non-Dutch	531.97 ± 10.0	532.37 ± 10.27	.86	
Parent Household				.762
Two-parent household	535.18 ± 9.40	533.20 ± 9.42	<0.001***	
Single-parent household	532.55 ± 9.49	531.07 ± 9.42	.20	
Household income				.101
Low	532.31 ± 9.68	531.26 ± 9.21	.17	
Intermediate	535.41 ± 9.11	533.70 ± 9.50	0.019*	
High	539.20 ± 7.81	535.63 ± 9.27	0.005**	
Treated concurrently with				
Asthma medication	537.10 ± 8.52	535.64 ± 8.94	.591	

Abbreviations: n, sample size; M, mean; SD, standard deviation

*Significant at .05 level**Significant at the 0.01 level

***Significant at the 0.001 level

Results of the crude and adjusted mean Cito-test scores controlled for potential effects of sex, ethnicity, parent household and household income are presented in Table 3.

**Table 3:** Crude and adjusted mean Cito-test scores for different methylphenidate treatment characteristics

1. MPH treatment	School performance			
	Crude M ± SD	Adjusted^a^ M ± SE	n	*p* value
Current users	532.80 ± 9.44	532.58 ± .48	377	<0.001***
Never users	534.71 ± 9.47	534.72 ± .11	7295	
2. Past versus current users	School performance			
	Crude M ± SD	Adjusted^a^ M ± SE	n	*p* value
Current users (*n* = 377)	532.80 ± 9.44	532.58 ± .48	377	.470
Past users (*n* = 64)	531.52 ± 10.70	531.67 ± 1.15	64	
3. Dosage of MPH treatment	School performance			
	Crude M ± SD	Adjusted^a^ M ± SE	n	*p* value
≤ 0.999 NDDD/Day	533.17 ± 9.60	533.16 ± .66	202	.498
> 0.999 NDDD/Day	532.44 ± 9.28	532.45 ± .81	132	
4. Start of MPH treatment	School performance			
	Crude M ± SD	Adjusted^a^ M ± SE	n	*p* value
Late start ≤12 months	536.37 ± 8.62	536.94 ± 1.51	38	.004**
Early start >12 months	532.40 ± 9.46	532.33 ± .50	339	
5. Concurrent treatment	School performance			
	Crude M ± SD	Adjusted^a^ M ± SE	n	*p* value
Only MPH	532.72 ± 9.45	532.70 ± .497	347	.937
MPH + AP	532.47 ± 9.71	532.87 ± 2.13	19	
Only asthma medication	537.10 ± 8.52	536.90 ± .865	100	.845
MPH + asthma medication	535.64 ± 8.94	537.49 ± 2.86	11	

Abbreviations: M, mean; SD, standard deviation; SE, standard error; n; sample size*Significant at the .05 level

**Significant at the 0.01 level

***Significant at the 0.001 level

^a^Adjusted for sex, ethnicity, parent household, and household income

After adjustments for the potential confounders, the means of Cito-test scores still differed significantly between the current users and never users. The difference in test score between groups corresponds to a 4% difference when taking into account the score range and a small standardized difference between means (Cohen’s d of 0.20). The adjusted mean estimates remained similar to the crude mean Cito-test scores indicating limited influence of confounding.

In the second part of the table the comparison is made between past and current users of methylphenidate. Both crude and adjusted analyses show no significant difference between both groups. All the covariates, sex, ethnicity, parent household, and household income, had significant influence on the outcome of the mean Cito-test scores.

When comparing the different dose regimens of methylphenidate (≤0.999 & >0.999 NDDD/Day) no significant difference was shown in both the crude and the adjusted models.

Compared with late starters (536.94 ± 1.51) we found significantly lower Cito-test scores for the early starters (532.33 ± .50) with a small standardized difference between means of the crude data (Cohen’s d of 0.43). The adjusted mean of the effect of start treatment on Cito-test scores changed only minimally when we included the covariates, indicating only negligible confounding by these variables. The average Cito-test scores did not differ significantly between early and late starters among the covariates ethnicity and parent household. The adjusted mean Cito-test scores reported remained nearly the same when controlling simultaneously for all covariates except for the methylphenidate + asthma medication group. We found no significant differences in Cito-test scores when comparing the different concurrent medication groups. Average Cito-test scores differed significantly only among the covariate sex for the concurrent treatment with antipsychotics.

## Discussion

This study shows that methylphenidate users have a lower school performance compared to children who have no history of ADHD medication. Furthermore, our results indicate that early starters of methylphenidate treatment have significantly lower school performance than children starting later with the treatment. Finally, past treatment compared to current treatment of methylphenidate, different dosage of methylphenidate and concurrent treatment with antipsychotic or asthma medication all appear to not be related to school performance.

In line with the previously established association between ADHD and poor school performance we found that children using methylphenidate had lower school performance compared with those who never used methylphenidate [2–5, 20]. This indicates that methylphenidate treatment does not normalize school performance in our study population, however, it may be questioned whether the difference is clinically relevant which is reflected by the small standardized differences between school performances of the different treatment groups. To what extent methylphenidate treatment may still have contributed to improvement of school performance cannot be concluded from our observational data given the lack of a non-treated ADHD group. The absent association between methylphenidate dosage and school performance suggests no major effects. Grizenko et al.(2006) found that children with ADHD and comorbid learning disability tended to respond more poorly to methylphenidate [21]. It could be that normalization of school performance only occurred in children without comorbid learning disabilities and therefor lowered the result of our analyses.In our study, we found that early starters of methylphenidate treatment had significantly lower school performance than children starting later with the treatment. It may be important to emphasize that late starters had a higher average Cito-test score than the national average of the Netherlands, which is around 535 [http://www.cito.nl/onderzoek%20en%20wetenschap/achtergrondinformatie/primair_speciaal_onderwijs/eindtoets_onderzoek_achtergrond]. When comparing early start of treatment with late start of treatment, the short-term effects of methylphenidate may explain these findings, whereas the long-term effects seem less effective. This is in line with existing studies that demonstrated the efficacy of short-term stimulant treatment [22–24]. With the correction for confounding factors it is not likely that we could be dealing with a subgroup of highly intellectual children.

However, we cannot rule out bias due to different disease characteristics between children starting treatment late and early. These differences may introduce bias to comparison, i.e., confounding by indication. It might be expected that children with severe symptoms and more persistent school problems start treatment earlier than those with less severe symptoms. With regard to the subtypes of ADHD, children with hyperactive/impulsive symptoms might be recognized and treated earlier than those who have predominantly inattentive symptoms, because the hyperactivity/impulsive behavior is more disruptive in the classroom [17]. A previous study in Iceland also investigated the difference in start of treatment [17]. Although they reported a significantly lower risk of decline in school performance between ages 9 and 12 with earlier start of stimulants. Within our study the influence of treatment on school performance over time could not be examined, as we looked at the performance on one test.

Comparing current users and past users showed no difference on school performance, which strengthens the conclusion that methylphenidate treatment either is losing its long-term effect or that the past users are a group with less severe ADHD problems. Because there was no difference measured in the different dosage of treatment groups it is less likely that the early starters of methylphenidate consist of children with more severe ADHD. It is not likely that missing data of dosage information in 11.4% of the children affected these results, because the missing data of the dosage information is caused by the incorrect or missing registration of this information by the pharmacist.

With respect to potential confounding by co-morbidities, on which we also lack direct information, we attempted to capture co-existing psychiatric disorders by accounting for concurrent antipsychotic use.However, we were unable to identify an association between school performance and use of antipsychotics This is in line with other studies [25, 26].

In addition, we investigated the difference in school performance among children who were concurrently treated with asthma medication and only asthma medication users. Again, no significant difference was found.

In the current study, we employed the unique setting by linking dispensed prescription records with school records to assess the effect of methylphenidate use in childhood on primary school performance. A strength of this study was, that we measured drug use by using pharmacy records. This minimized the risk of recall bias, often associated with survey data, and selection bias associated with use of localized community data. Another strong aspect is the possibility to track children over time until the Cito-test was taken [16]. Furthermore, our objective outcome measure was standardized test scores and because of this we were able to compare the scores from year to year despite differences in the difficulty of the tests.

The study also had several limitations. The biggest limitation is the fact that we were not able to take baseline ADHD severity into account. It is not unreasonable to assume that more severely affected children would have been identified at earlier ages and therefore started earlier on methylphenidate. In the absence of any controls, it is impossible to know how these children would have performed on the Cito-test had they not received methylphenidate.

In addition, there was no information about the magnitude of potential bias created by other possible medication use, family factors and additional school services, which could have over- or underestimated the actual difference in overall school performance between methylphenidate users and never users. Since the IADB.nl database contains only records of dispensed medication it is not possible to measure the actual medication use. Non-adherence could have led to an overestimation of the actual use of methylphenidate.

Finally, in this study we controlled for several confounders. Our data indicate that methylphenidate use and school performance is somewhat different across boys and girls. Boys performed better on Cito-test than girls. Additionally, in the multivariable analysis the interaction between methylphenidate use and child’s sex was significant. This is in line with existing studies, which reveal that boys are three to four times more likely than girls to be diagnosed with ADHD based on studies of community samples. In clinically referred samples the sex differences is as high as five- to nine-fold [27–29]. We assume that methylphenidate use does not have sex-specific effects on school performance and it is therefore likely that ADHD plays a major role. The other possible confounders, which we included in our multivariable analysis, did not show significant effects.

In the subgroup analysis, it was remarkable that in both the methylphenidate users and the never users group the school performance was better as household income was higher. On average, children with a high household income scored nearly 7 point higher in the never users group and approximately 4 points higher in the current users group compared to the lowest income bracket. This is consistent with a previous study that is also conducted with Cito-test scores by researchers of the CBS. [http://www.cbs.nl/en-GB/menu/themas/onderwijs/publicaties/artikelen/archief/2013/2013-3815-wm.htm].

It should also be noted that there might be some unmeasured confounding as a result of our limitations. Taking into account potential clustering of children within schools and/or classrooms could have prevented part of the unmeasured confounding, but such clustered analysis was not possible because such identifying information was for privacy reasons not available in the database. Potentially, the severity of ADHD symptoms and comorbid learning disabilities could have influenced the school performance. It is known from literature that children with comorbid learning disabilities respond poorer to stimulant treatment, and this should be taken into account in a future study [21]. We also lack information about non-pharmacological treatment like concurrent behavior therapy and additional school services. However, from literature we know that concurrent behavioral therapy provides only modest advantages compared with drug treatment alone [30]. Thus, this may not be of major concern.

We suspect that children who received special educational services had better outcomes than children without such services. In a further study this effect could be better explored, while also taking into account the perspectives of the teachers.

Although our study showed an association between various parameters of methylphenidate use and school performance, it must be noted that without a clear comparator group and using only correlational data no conclusions can be drawn about cause and effect of methylphenidate use and school performance.

Importantly we need prospective, controlled and large-scaled studies on the long term to evaluate whether short term or chronic methylphenidate treatment will improve school performance of children with ADHD.

## Conclusion

In this retrospective descriptive study, earlier start of methylphenidate treatment was associated with a lower school performance than children starting later with the treatment which could indicate a limited effect of long term treatment or a more strongly affected group of early starters. Our study also indicates that children using methylphenidate still perform less at school compared to their peers. More study is however warranted to unravel why children who are methylphenidate treated do not score similar as their peers, especially when treatment starts early.

## Abbreviations

ADHD: Attention-Deficit/Hyperactivity Disorder
IADB: InterAction Database
NDDD: Number of defined daily dose

## References

[1] Polanczyk GV, Willcutt EG, Salum GA, Kieling C, Rohde LA (2014). ADHD prevalence estimates across three decades: an updated systematic review and meta-regression analysis. Int J Epidemiol.

[2] Barbaresi WJ, Katusic SK, Colligan RC, Weaver AL, Jacobsen SJ (2007). Long-term school outcomes for children with attention-deficit/hyperactivity disorder: a population-based perspective. J Dev Behav Pediatr.

[3] Loe IM, Feldman HM (2007). Academic and educational outcomes of children with ADHD. J Pediatr Psychol.

[4] Polderman TJ, Boomsma DI, Bartels M, Verhulst FC, Huizink AC (2010). A systematic review of prospective studies on attention problems and academic achievement. Acta Psychiatr Scand.

[5] Barry T, Lyman R, Klinger L (2002). Academic underachievement and attentiondeficit/hyperactivity disorder: the negative impact of symptom severity on school performance. J Sch Psychol.

[6] Sonuga-Barke EJ, Brandeis D, Cortese S, Daley D, Ferrin M, Holtmann M, Stevenson J, Danckaerts M, van der Oord S, Dopfner M, Dittmann RW, Simonoff E, Zuddas A, Banaschewski T, Buitelaar J, Coghill D, Hollis C, Konofal E, Lecendreux M, Wong IC, Sergeant J (2013). European ADHD guidelines group: Nonpharmacological interventions for ADHD: systematic review and meta-analyses of randomized controlled trials of dietary and psychological treatments. Am J Psychiatry.

[7] Trip AM, Visser ST, Kalverdijk LJ, de Jong-van den Berg LT (2009). Large increase of the use of psycho-stimulants among youth in the Netherlands between 1996 and 2006. Br J Clin Pharmacol.

[8] Storebo OJ, Krogh HB, Ramstad E, Moreira-Maia CR, Holmskov M, Skoog M, Nilausen TD, Magnusson FL, Zwi M, Gillies D, Rosendal S, Groth C, Rasmussen KB, Gauci D, Kirubakaran R, Forsbol B, Simonsen E, Gluud C (2015). Methylphenidate for attention-deficit/hyperactivity disorder in children and adolescents: Cochrane systematic review with meta-analyses and trial sequential analyses of randomised clinical trials. BMJ.

[9] Banaschewski T, Gerlach M, Becker K, Holtmann M, Dopfner M, Romanos M (2016). Trust, but verify. The errors and misinterpretations in the Cochrane analysis by O. J. Storebo and colleagues on the efficacy and safety of methylphenidate for the treatment of children and adolescents with ADHD. Z Kinder Jugendpsychiatr Psychother.

[10] Wigal SB, Wigal T, Schuck S, Brams M, Williamson D, Armstrong RB, Starr HL (2011). Academic, behavioral, and cognitive effects of OROS(R) methylphenidate on older children with attention-deficit/hyperactivity disorder. J Child Adolesc Psychopharmacol.

[11] Wigal SB, Childress AC, Belden HW, Berry SA (2013). NWP06, an extended-release oral suspension of methylphenidate, improved attention-deficit/hyperactivity disorder symptoms compared with placebo in a laboratory classroom study. J Child Adolesc Psychopharmacol.

[12] Hammerness P, Fried R, Petty C, Meller B, Biederman J (2014). Assessment of cognitive domains during treatment with OROS methylphenidate in adolescents with ADHD. Child Neuropsychol.

[13] Hechtman L, Abikoff H, Klein RG, Weiss G, Respitz C, Kouri J, Blum C, Greenfield B, Etcovitch J, Fleiss K, Pollack S (2004). Academic achievement and emotional status of children with ADHD treated with long-term methylphenidate and multimodal psychosocial treatment. J Am Acad Child Adolesc Psychiatry.

[14] Marcus SC, Durkin M (2011). Stimulant adherence and academic performance in urban youth with attention-deficit/hyperactivity disorder. J Am Acad Child Adolesc Psychiatry.

[15] Prasad V, Brogan E, Mulvaney C, Grainge M, Stanton W, Sayal K (2013). How effective are drug treatments for children with ADHD at improving on-task behaviour and academic achievement in the school classroom? A systematic review and meta-analysis. Eur Child Adolesc Psychiatry.

[16] Visser ST, Schuiling-Veninga CC, Bos JH, de Jong-van den Berg LT, Postma MJ (2013). The population-based prescription database IADB.Nl: its development, usefulness in outcomes research and challenges. Expert Rev Pharmacoecon Outcomes Res.

[17] Zoega H, Rothman KJ, Huybrechts KF, Olafsson O, Baldursson G, Almarsdottir AB, Jonsdottir S, Halldorsson M, Hernandez-Diaz S, Valdimarsdottir UA (2012). A population-based study of stimulant drug treatment of ADHD and academic progress in children. Pediatrics.

[18] Schmitt J, Buske-Kirschbaum A, Roessner V (2010). Is atopic disease a risk factor for attention-deficit/hyperactivity disorder? A systematic review. Allergy.

[19] Hak E, de Vries TW, Hoekstra PJ, Jick SS (2013). Association of childhood attention-deficit/hyperactivity disorder with atopic diseases and skin infections?A matched case-control study using the general practice research database. Ann Allergy Asthma Immunol.

[20] Wilens TE, Biederman J, Brown S, Tanguay S, Monuteaux MC, Blake C, Spencer TJ (2002). Psychiatric comorbidity and functioning in clinically referred preschool children and school-age youths with ADHD. J Am Acad Child Adolesc Psychiatry.

[21] Grizenko N, Bhat M, Schwartz G, Ter-Stepanian M, Joober R (2006). Efficacy of methylphenidate in children with attention-deficit hyperactivity disorder and learning disabilities: a randomized crossover trial. J Psychiatry Neurosci.

[22] Abikoff H, Hechtman L, Klein RG, Weiss G, Fleiss K, Etcovitch J, Cousins L, Greenfield B, Martin D, Pollack S (2004). Symptomatic improvement in children with ADHD treated with long-term methylphenidate and multimodal psychosocial treatment. J Am Acad Child Adolesc Psychiatry.

[23] Vitiello B (2001). Long-term effects of stimulant medications on the brain: possible relevance to the treatment of attention deficit hyperactivity disorder. J Child Adolesc Psychopharmacol.

[24] Biederman J, Faraone SV (2005). Attention-deficit hyperactivity disorder. Lancet.

[25] Barbaresi WJ, Katusic SK, Colligan RC, Weaver AL, Jacobsen SJ (2007). Modifiers of long-term school outcomes for children with attention-deficit/hyperactivity disorder: does treatment with stimulant medication make a difference? Results from a population-based study. J Dev Behav Pediatr.

[26] Frick PJ, Kamphaus RW, Lahey BB, Loeber R, Christ MA, Hart EL, Tannenbaum LE (1991). Academic underachievement and the disruptive behavior disorders. J Consult Clin Psychol.

[27] Barkley RA (2014). Attention-deficit hyperactivity disorder: a handbook for diagnosis and treatment.

[28] Gaub M, Carlson CL (1997). Gender differences in ADHD: a meta-analysis and critical review. J Am Acad Child Adolesc Psychiatry.

[29] Gershon J (2002). A meta-analytic review of gender differences in ADHD. J Atten Disord.

[30] Sonuga-Barke EJ, Brandeis D, Cortese S, Daley D, Ferrin M, Holtmann M, Stevenson J, Danckaerts M, van der Oord S, Dopfner M, Dittmann RW, Simonoff E, Zuddas A, Banaschewski T, Buitelaar J, Coghill D, Hollis C, Konofal E, Lecendreux M, Wong IC, Sergeant J, European ADHD Guidelines Group (2013). Nonpharmacological interventions for ADHD: systematic review and meta-analyses of randomized controlled trials of dietary and psychological treatments. Am J Psychiatry.

